# Cellular Energetics of Mast Cell Development and Activation

**DOI:** 10.3390/cells10030524

**Published:** 2021-03-02

**Authors:** Ryan P. Mendoza, Dylan H. Fudge, Jared M. Brown

**Affiliations:** Department of Pharmaceutical Sciences, Skaggs School of Pharmacy and Pharmaceutical Sciences, University of Colorado Anschutz Medical Campus, Aurora, CO 80016, USA; ryan.mendoza@cuanschutz.edu (R.P.M.); dylan.fudge@cuanschutz.edu (D.H.F.)

**Keywords:** mast cells, energy metabolism, glycolysis, mitochondrial respiration, oxidative phosphorylation, activation, development, mastocytosis, mast cell activation syndrome (MCAS), mast cell metabolism

## Abstract

Mast cells are essential first responder granulocytes in the innate immune system that are well known for their role in type 1 immune hypersensitivity reactions. Although mostly recognized for their role in allergies, mast cells have a range of influences on other systems throughout the body and can respond to a wide range of agonists to properly prime an appropriate immune response. Mast cells have a dynamic energy metabolism to allow rapid responsiveness to their energetic demands. However, our understanding of mast cell metabolism and its impact on mast cell activation and development is still in its infancy. Mast cell metabolism during stimulation and development shifts between both arms of metabolism: catabolic metabolism—such as glycolysis and oxidative phosphorylation—and anabolic metabolism—such as the pentose phosphate pathway. The potential for metabolic pathway shifts to precede and perhaps even control activation and differentiation provides an exciting opportunity to explore energy metabolism for clues in deciphering mast cell function. In this review, we discuss literature pertaining to metabolic environments and fluctuations during different sources of activation, especially IgE mediated vs. non-IgE mediated, and mast cell development, including progenitor cell types leading to the well-known resident mast cell.

## 1. Introduction

### 1.1. Mast Cells

Mast cells are important effector cells in the innate immune system. Mast cells release a variety of mediators following recognition of foreign substances and endogenous damage associated molecular patterns (DAMPs) [1]. This cell type is mostly recognized for its role in type 1 immune hypersensitivity response (IgE mediated allergies); however, as first responders they are implicated in the activity of many systems within the body including proper gastrointestinal, pulmonary, and neuronal functioning [2,3,4]. Further, their central role in immune cell recruitment/activation means that any prolonged mast cell dysfunction manifests as pathologies across the body. Diseases based on mast cell dysfunction are categorized as mast cell activation syndromes (MCAS) and include a wide range of etiologies and symptoms [5]. In addition to MCAS, mast cells can play a role in many other diseases including mastocytosis, asthma, multiple sclerosis (MS), and gut pathologies such as ulcerative colitis and Crohn’s disease [6,7,8,9,10,11,12]. To identify early diagnosis methods and develop therapeutics for these diseases, the mechanisms involved in mast cell development and activation must be better understood.

Most research focuses on the high affinity IgE receptor (FcεR1), which requires sensitization with IgE and a secondary exposure to the agonist to induce activation. However, there are many mast cell receptors that do not need IgE to activate and are often referred to as “non-IgE receptors” [13]. Non-IgE receptors only require a single exposure to initiate non-IgE mediated activation. Examples of non-IgE receptors on mast cells are Toll-like receptors (e.g., TLR4), G-protein coupled receptors (e.g., C3aR), and alarmin receptors (e.g., IL-1R) [14,15,16]. This and the sheer number of different non-IgE receptors warrants more research into this category of activation to fully elucidate the function of mast cells. There are two general phases of activation: early phase (degranulation) and late phase. These cells can respond immediately with early phase degranulation by releasing the contents of their preformed granules, which include a variety of proteases, chemokines, and cytokines. One unique feature of mast cells is their ability to respond to stimuli either by slowly releasing (piecemeal degranulation) or rapidly releasing (anaphylactic degranulation) their prepackaged mediators at once [17]. They also perform late phase de novo synthesis and release of other inflammatory mediators over the span of 6–24 h after activation. These include cytokines, growth factors, and lipid mediators such as arachidonic acid metabolites like leukotrienes (LT) and prostaglandins (PG). The central function of mast cells is to both identify danger and appropriately prime an immune response to the perceived danger. Both of these aspects are affected either directly or indirectly by the process of cellular energy metabolism pathways such as glycolysis and oxidative phosphorylation. This review will present the current understanding on how energy metabolism contributes to the development and essential functions of mast cells.

### 1.2. Energy Metabolism

Cellular metabolism includes all metabolic pathways within the cell that involve producing energy or building complex molecules to be broken down for energy production in the future. The act of breaking down molecules to oxidize and produce energy is defined as catabolism. This involves classical metabolic pathways such as glycolysis, mitochondrial respiration, and fatty acid oxidation. In contrast, anabolism involves processes that utilize energy to build up complex molecules for future use. Examples of anabolic metabolism are gluconeogenesis and the pentose phosphate pathway (PPP). Most research into mast cell metabolism focuses on the role of the central energy producing catabolic pathways glycolysis and mitochondrial respiration.

Glycolysis is dependent on uptake of extracellular glucose through glucose transporters (GLUT) and results in either the export of lactic acid to extracellular space or shunting of pyruvate molecules to the mitochondria, in the form of acetyl-CoA, for use in the tricarboxylic acid cycle (TCA cycle)/Krebs cycle. ‘Mitochondrial respiration’ is comprised of the full process of the TCA cycle production of NADH and FADH_2_ for use in the electron transport chain (ETC) for oxidative phosphorylation of ADP to ATP in Complex V (i.e., ATP Synthase). This mechanism results in complete metabolism of nutrients to CO_2_ and H_2_O. In addition to pyruvate derived from glycolysis, mitochondrial respiration oxidizes glutamine and products of fatty acid oxidation. Fatty acid oxidation (FAO) is the progressive shortening of long and short chain fatty acids to acetyl-CoA, which can be utilized in the TCA cycle. Glutamine is transported into the mitochondria by glutamine transporters (SLC) and converted to α-Ketoglutarate for use in the TCA cycle. A requirement for mitochondrial respiration to function optimally with minimal production of unwanted reactive oxygen species (ROS) and maximal production of ATP is a healthy mitochondrion. Proper mitochondrial fitness can be maintained through the endless fission-fusion events occurring between mitochondria within the cell along with mitophagy. Both mechanisms eliminate damaged mitochondria from the cell.

There are advantages and disadvantages to either of these central arms of catabolic metabolism. As mentioned, mitochondrial respiration can utilize several more nutrients to produce energy than glycolysis. Importantly, oxidative phosphorylation produces 32 ATP per glucose molecule oxidized while glycolysis only produces 2 ATP [18]. However, glycolysis can oxidize many glucose molecules at once, providing a large energy burst that oxidative phosphorylation cannot match. Also, respiration requires oxygen (mitochondrial respiration is often termed aerobic metabolism) whereas glycolysis can be anaerobic, allowing utilization in hypoxic situations. Glycolysis also supplies intermediates that are essential for the anabolic pentose phosphate pathway (PPP) which is involved in fatty acid, ribose, and amino acid synthesis. PPP is also important in NADP^+^/NADPH homeostasis and provides DNA precursors for proliferation [19,20]. Therefore, both pathways are needed to act in homeostasis for proper energy production for cellular function and development.

This review will cover literature pertaining to mast cell energy metabolism during both activation and development. The regulation of packaging and mediator release to the extracellular environment during activation requires an adaptable metabolism to shift between the dynamic energetic demands of the cell. The balance of energy in mature mast cells presents its own difficulties to oblige the diverse metabolic demands when transitioning between the inactive and specific active states—IgE mediated vs. non-IgE mediated; early phase vs. late phase. The plastic metabolic nature of mast cell activation will be covered in the initial portions of this review. Further, there are a variety of metabolic changes occurring during the development of a mast cell, which have effects extending beyond merely providing energy for the cell. The latter focus of this review is on the metabolic transformations occurring to convert hematopoietic stem cells to mast cell progenitors to mature mast cells and how these energy changes potentially influence cell fate. Research has only just begun to reveal the enormous role cellular metabolism plays in both mast cell development and activation.

## 2. Activation

Mature mast cells can respond to a variety of endogenous and exogenous compounds through two general categories of activation: early phase degranulation and late phase activation. Mast cell activation is commonly defined based on receptor interactions: either through FcεR1 (IgE mediated) or any other receptors (non-IgE mediated). Research into the metabolic changes during, and as a result of, different mechanisms of activation is not extensive. It has been established that basal mast cells perform both glycolysis and mitochondrial respiration at levels that can be modified uniquely depending on the mechanism of activation [21,22]. This section of the review will focus on those changes that occur during mast cell activation.

### 2.1. IgE Mediated Activation

IgE mediated degranulation is the most researched mechanism of mast cell activation (Table 1). Despite this, little is known about its metabolic requirements or post-activation effects on cellular metabolism. As previously mentioned, this mechanism of degranulation requires sensitization with IgE antibody prior to activation. There has been minimal work performed exploring how this sensitization step modulates metabolism in mast cells. However, priming FcεR1 with IgE antibody likely does induce metabolic shifts and is an important topic of future research. Older studies, mostly performed by the Chakravty laboratory, focused on deciphering the metabolic mechanism of the secondary exposure activation utilizing rat peritoneal mast cells. These first established that IgE mediated degranulation increases lactate export in this cell type, indicating an increase in glycolysis. Further, they were able to identify a positive correlation between histamine release and degree of glycolytic metabolic stimulation [23]. Chakravty also established that histamine release from antigen stimulation was inhibited by pre-treatment with 2-deoxyglucose (2-DG) [24]. 2-DG is an inhibitor of glycolysis that acts by binding to hexokinase, the first enzyme in GLUTs. It binds with a higher affinity than glucose but is not metabolized further than hexokinase, essentially blocking this enzyme from processing glucose molecules. Correlating this pre-treatment to lower granule release shows a role for glycolysis in IgE mediated degranulation.

Chakravty also explored the role of mitochondrial respiration/oxidative phosphorylation during degranulation. He demonstrated that respiration in rat peritoneal mast cells, measured by oxygen consumption, was markedly increased 15–20 min post activation [25]. This increase was much more significant in media lacking glucose compared to in the presence of glucose. It appears when mast cells cannot utilize glycolysis (from absence of glucose), as oxidative phosphorylation then upregulates to compensate for a lack of energy production from the glycolytic arm. This is strong evidence that mast cells upregulate specific metabolic pathways based on nutrients available during IgE mediated degranulation.

Initial studies into mast cell activation dependent metabolism shifts also explored the role of ATP during degranulation. It was quickly established that there was a positive correlation between ATP levels in mast cells and the amount of histamine released during antigen stimulated activation [26]. Interestingly, inhibiting glycolysis or mitochondrial respiration (separately) did not lead to significant changes in ATP production but did lead to inhibition of histamine release [26,27]. In this experiment, glycolysis was inhibited by 2-DG, as described above, and respiration was inhibited by oligomycin. Oligomycin is an ATP Synthase inhibitor that acts by blocking the proton channel (Fo subunit) essential for oxidative phosphorylation of ADP to ATP. This appears to be a conundrum, since these results showed that ATP correlates to histamine release but blocking specific pathways will change histamine release without modulating ATP production. From these experiments, mast cells appear to be able to compensate for ATP production from downregulation of specific metabolic pathways but not compensate in granule release during IgE mediated degranulation. This suggests that this mechanism of activation prefers glycolysis or mitochondrial respiration independently of ATP and that perhaps intermediates of the pathways play a larger role in activation than simply producing ATP. These early findings by the Chakravty group became the basis of our knowledge for mast cell metabolism.

Mast cell research evolved and began utilizing cell lines other than isolated rat peritoneal cells. Many groups began researching cellular metabolic function with murine bone-marrow derived mast cells (BMMCs). When these primary mast cells are cultured in media that contains varying concentrations of D-glucose, there were significant changes in BMMC metabolic function and activation. After long-term exposure to high glucose, there were increases in IgE mediated degranulation, measured by β-hexosaminidase release, and late phase activation, measured by leukotriene C4 and prostaglandin D2 release. This occurred without any changes in surface expression of FcεR1 or intracellular ATP levels [28]. In another cell line, immortalized RBL-2H3 (Rat Basophilic Leukemia cells), it was demonstrated that M2-type pyruvate kinase can interact directly with the gamma chain of the FcεR1 in an inhibitory fashion [29]. Activation through the high-affinity IgE receptor will downregulate this pyruvate kinase and allow degranulation to occur. If M2-type pyruvate kinase continues interacting with the receptor, there will be no activation even with proper stimulation. Pyruvate kinase is a later intermediate in the glycolytic pathway, so these results establish an interaction between glycolytic substrates and degranulation pathway. This may also be the mechanism through which glucose levels can affect activation: higher glucose leads to less free pyruvate kinase and less interaction with FcεR1, resulting in higher activation.

To further demonstrate the importance of glycolysis, mast cells were exposed to nitric oxide (NO) which is known to inhibit IgE mediated degranulation. This group showed NO will nitrate a tyrosine on aldolase A to reduce its function in human mast cell lines LAD2 and HMC-1. Aldolase A is an enzymatic intermediate of the glycolytic pathway that catalyzes its substrate fructose-1,6-bisphosphate (FBP) to glyceraldehyde 3-phosphate (G3P) and dihydroxyacetone phosphate (DHAP). Interestingly, inhibition of this enzyme did not significantly change ATP production, but did increase the AMP:ATP ratio, thereby potentially incorporating AMP-activated pyruvate kinase into the mechanism. Adding to the surprising results, inhibiting this enzyme led to increased glycolysis in these cell types measured by increases in lactate and pyruvate [30]. This reduction in aldolase A also led to higher levels of fructose-1,6-bisphosphate, which was demonstrated to inhibit IgE mediated mast cell degranulation and could explain the decrease in β-hexosaminidase observed. These seemingly confounding results confirms the complexity of metabolic pathways in mast cells and adds to the credence that metabolic pathway intermediates are perhaps more important to activation than ATP production.

Mitochondrial respiration has also been explored during IgE mediated mast cell degranulation. It was established that antigen stimulated activation of RBL-2H3 and LAD2 mast cell lines will induce translocation of mitochondria from perinuclear to exocytosis sites, strongly hinting that mitochondria have a role in this cellular function [31]. This occurred during early phase degranulation as well as late phase production and secretion of TNFa. When further explored, it was discovered that IgE mediated degranulation significantly increased mitochondrial respiration in a STAT3-dependent manner in RBL-2H3, LAD2, and BMMC cells lines [32]. When phosphorylation of mitochondrial STAT3 or ATP production from oxidative phosphorylation was inhibited, there was inhibition of mast cell degranulation. The role of pyruvate dehydrogenase (PDH), an intermediate that can regulate the TCA cycle by catalyzing the conversion of pyruvate to acetyl-CoA, was also explored. It was shown that inhibiting PDH, and therefore mitochondrial respiration, led to decreased IgE mediated degranulation and decreased secretion of TNFa and IL-6 in RBL-2H3 and BMMCs [33]. Inhibition was also shown to result in lower histamine levels in the mice of lungs sensitized and exposed to allergen in vivo. PDH is regulated by a mitochondrially located transcription factor—the microphthalmia transcription factor (MITF). When this transcription factor was overexpressed, PDH function was decreased which led to higher pyruvate levels, lower oxygen consumption, and lower ATP production. This demonstrates the important role for both glycolysis and mitochondrial respiration homeostasis for proper IgE mediated mast cell activation. However, non-IgE mediated activation has the potential for unique metabolic responses compared to IgE mediated activation.

### 2.2. Non-IgE Mediated Activation

While most research on general mast cell function and metabolism has been performed based on IgE mediated stimulation, there are many other elements that lead to non-IgE mediated activation (Table 2). Chakravty et al. did similar work to establish basal knowledge of how cells respond to non-IgE stimuli. They mostly utilized the classical mast cell agonist compound 48/80, an exogenous polymer formed by condensing N-methyl-p-methoxyphenethylamine with formaldehyde. This agonist is now known to activate mast cells through the Mas-related G protein coupled receptor X1/X2 (MRGPRX1/X2) in humans, B2 analog (MRGPRB2) in mice, and B3 (MRGPRB3) analog in rats [34,35,36]. They showed that peritoneal mast cells will upregulate glucose metabolism undergoing this mechanism of degranulation [23]. It was also demonstrated that this mechanism will significantly upregulate the pentose phosphate pathway (PPP) by up to 80% [37]. It was indicated that PPP stimulation may be for post-exocytosis regenerative purposes. They also researched the role of ATP in activation similar to experiments described in relation to IgE mediated degranulation above. There was a positive correlation between histamine release and ATP levels [26], showing that both IgE mediated and non-IgE mediated activation are likely dependent on ATP production and utilization. These results were confirmed years later using 1H NMR to show histamine release from compound 48/80 was correlated to increased lactate release, linking this mechanism of degranulation with glycolysis [38].

Like compound 48/80, IL-33 is a well-known mast cell agonist. IL-33 increases both glycolysis and oxidative phosphorylation when activating BMMCs, measured by extracellular acidification rate and oxygen consumption using Seahorse technology [39]. Blocking glycolytic function with 2-DG will significantly inhibit the mast cells ability to release cytokines following IL-33 stimulation including TNFa, IL-6, and CCL2. This pre-treatment also reduces mast cell ability to recruit neutrophils in vivo, providing a rare direct link between the cellular metabolism and cell-recruiting ability of mast cells. This effect is reversed with the introduction of ATP leading authors to suggest ATP availability may be responsible for this inhibition. However, extracellular ATP can independently activate mast cells, to be discussed below. This paper also employed an agonist of AMP-activated pyruvate kinase, which often has been shown to shift metabolic activity from glycolysis to oxidative phosphorylation, and showed this also reduced IL-33 activation, mirroring glycolytic inhibition demonstrated with 2-DG. LPS exposure to BMMCs and peritoneal mast cells leads to suppression of cytokine production including IL-6 and CCL2 [40]. This inhibitory effect was further increased by exposure to lactic acid. Lactic acid is an end product of glycolysis and is likely acting in a negative feedback manner to decrease glucose uptake and lactate export. This effect was re-created with glycolytic inhibitors, which provides more evidence that LPS may be downregulating glycolysis to suppress non-IgE mediated late phase activation. Authors were able to rescue this effect by dosing cells with ATP and concluded, like in the studies above, that ATP availability is the important part of mast cell function. This could potentially be the case, but it is possible that ATP is acting independently to activate mast cells through surface receptors.

Extracellular ATP can activate mast cells though the interaction with purinergic (P2) receptors including P2X and P2Y [41]. This brings into question the methodology of dosing cells with ATP to counteract ATP production from inhibiting metabolic pathways. It also adds to the intricacy of the role these pathways and energy production plays in mast cell activation. This activation appears to be synergistic with IgE-mediated degranulation and co-exposure of BMMCs to extracellular adenosine and antigen will induce degranulation at concentrations that normally would lead to no response [42]. This interaction and redundancy of non-IgE and IgE pathways demonstrates the complexity of mast cell response and the difficulty in fully elucidating these mechanisms.

### 2.3. Summary of Energetic Demands during Mast Cell Activation

It is apparent that mast cells can modify both glycolytic function and mitochondrial respiration/oxidative phosphorylation during activation. Further, it is shown these pathways will change differently depending on the source of mast cell activation and that blocking specific metabolic pathways will have varying effects on cell function. The limited literature suggests that perhaps IgE mediated activation may be more dependent on proper mitochondrial function and non-IgE mediated activation on glycolysis in comparison. However, this could be a result of most non-IgE mediated based research almost exclusively exploring glycolysis. A central theme throughout all mechanisms of activation is that ATP availability and usage is an important endpoint for cellular function. Increasing ATP availability was shown multiple times to rescue inhibition of mast cell function induced by blocking metabolic pathways. However, these results may be misinterpreted because ATP can induce activation by itself. Also, even with no changes in intracellular ATP there can be changes in mast cell function based on specific metabolic pathway utilization. Therefore, it is possible that intermediates of the metabolic pathways may play a larger role in proper mast cell function than simply being part of the mechanism that produces ATP.

There is a significant lack of literature regarding metabolic changes during activation. Most of the studies exploring the basic facets of metabolism were performed during 1960–1980, so many of the techniques used are obsolete and need to be re-explored with modern assays and equipment. Further, mast cells have been shown to perform phagocytosis on invading pathogens to deal with the threat directly instead of recruiting other immune cells to the site of injury [43,44]. However, there is no research addressing metabolism shifts during this function which could provide an interesting comparison to changes during early and late phase mast cell activation. There is also a gap in research looking at non-IgE mediated mast cell degranulation, which can occur as commonly because IgE mediated and led to similar pathophysiological outcomes. For example, when an atopic dermatitis mouse model, a mast cell specific pathology, was co-exposed to allergen and the non-IgE agonist silver nanoparticles (AgNP), there were significantly worsened symptoms than allergen alone [45]. Also, IL-33 activation of mast cells in the lung plays a prominent role in the initiation and development of airway hyper-responsiveness and pulmonary inflammation [46,47,48]. Many common mast cell agonists activate through non-IgE receptors (e.g., adverse drug allergies, complement, and neuropeptides). Cellular energy metabolism appears to be a diverging mechanism between the two categories of activation and even between separate non-IgE mediated pathways.

Differences between this basic function of the cell could provide important clues into how and why mast cells are being activated, as well as provide novel diagnosis techniques for identifying source of anaphylaxis. There needs to be more research establishing basal mast cell metabolism, as well as comparing different non-IgE mechanisms to IgE mediated mechanisms. This energy consumption is also essential during the development of mast cells from hematopoietic and progenitor cell populations. Shifts in metabolism during development may eventually lead to differences in cell phenotype that affect cell function. This next part of the review will focus on what literature has reported on metabolic shifts during the phases of development to a mature mast cell.

## 3. Development

**Figure 1:**
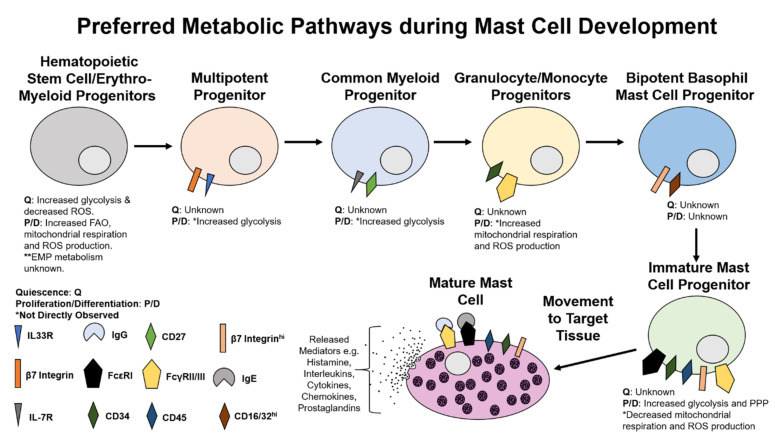
Preferred Metabolic Pathways during Mast Cell Development. Mast cells originate in the bone marrow as hematopoietic stem cells developing via multipotent progenitors, common myeloid progenitors, granulocyte/monocyte progenitors, immature mast cell progenitors prior to developing into fully mature mast cells in the target tissues. The metabolism at each developmental stage is described during both quiescence (Q) and differentiation/proliferation (P/D). Some of the preferred metabolic pathways were not directly observed (*) due to no direct experimental analysis having been performed on the metabolic pathways. The proposed metabolic pathways were determined through changes in known cellular signaling pathways and the effects these signaling pathways have on cellular metabolic pathways.

Mast cells are an integral part of the immune system originally believed to solely originate from a pluripotent hematopoietic lineage and further develop in the bone marrow [49]. However recent studies have found that while mast cell populations still originate from hematopoietic stem cells (HSCs) [50,51], there is evidence to suggest they may also originate from yolk sac derived erythro-myeloid progenitors (EMPs) [51]. Regardless of their beginning cell type immature mast cells will uniquely mature in the target tissue under the influence of growth factors and cytokines such as stem cell factor (SCF) [52,53]. The mediators released from mature mast cells can aid in the localization and development of immature mast cells to mature mast cells [54,55]. Mast cells begin their journey to maturity either in the bone marrow as hematopoietic stem cells (HSCs) [50] or as yolk sac EMPs to further differentiate into multipotent progenitors (MPP) [56]. Currently the pathway to mast cell maturity is still under debate, although it is most commonly believed that MPPs will then differentiate into common myeloid progenitors (CMPs). There is discord as to where the cell populations develop from this point, although traditional cell fate mapping has CMPs develop into either megakaryocyte/erythrocyte progenitors (MEPs) or granulocyte/monocyte progenitors (GMPs) [57], while it is commonly accepted that CMPs develop into GMPs [58]. GMPs will then mature into bipotent basophil/mast cell progenitors (BMCPs) prior to maturing into immature mast cells [59,60]. Immature mast cells will mature in the target tissue to mature mast cells through the aid of specific growth factors. One study suggests that the ambiguity with the mast cell progenitor pathway is due to development not following a traditional linear tree diagram but rather many different differentiated cells may develop from progenitor cells in nearby branches of the development tree [60]. This means that progenitors other than the ones described in this review may also mature into mast cells but there is less likelihood of them maturing to mast cells, as opposed to their traditional differentiation route along a tree diagram. For the purposes of this review, we only discuss the progenitor cells with the highest likelihood of maturing to mature mast cells. Little is currently known about the metabolic environment in these progenitor cell lines, which offers a niche to study the proliferation and differentiation of developing mast cells. Energy production is not static during these processes indicating a dynamic cellular metabolism in each progenitor cell type which may potentially be the effector to stimulate proliferation, differentiation, or quiescence (Figure 1).

### 3.1. Hematopoietic Stem Cells and Erythro-Myeloid Progenitors

The HSCs and EMPs are arguably some of the most dynamic cells in the body and the hematopoietic system. The constituents of the blood are constantly modulated to meet the temporal demands of the body. HSCs and EMPs have the capability to develop into ever increasingly committed progenitor cell lines including mast cells. EMPs are difficult to study largely due to their transient nature and the temporal overlap with HSC generation [61]. For this reason, there is minimal information regarding their metabolism and therefore this review will primarily focus on HSCs as the beginning cell ultimately leading to mature mast cells. HSCs robustly respond to stimuli requiring immediate and massive production of specific types of blood cells. To meet the blood system’s highly dynamic needs, HSCs require an equally dynamic and unique cellular metabolism. The metabolic profile for HSCs will adjust depending on the cellular state required (i.e., proliferation, quiescence, or differentiation). The journey through the metabolism of mast cells will begin with the distinctive metabolism of its original progenitor cell line, HSCs.

HSCs are often observed in a quiescence state to prevent unheeded growth and cellular damage from increased mitochondrial respiration and production of reactive oxygen species (ROS) [62]. During quiescence, HSCs have been shown to rely on glycolysis as opposed to aerobic metabolism to avoid unnecessary ROS production and meet the low energetic demands during this dormant state despite HSCs containing significantly higher concentrations of mitochondria when compared to other stem cells [63]. Additionally, there is work to support ROS driving cellular processes implying cellular metabolism (with ROS as a by-product) may have a multifaceted role with the ability to dictate cell fate in addition to being a byproduct of cellular energy production [64,65,66,67]. For example, when intracellular ROS is increased in HSCs (via unhealthy mitochondria), HSCs were forced to develop from a quiescence state driving differentiation and proliferation [50,68]. This indicates that ROS may act as a second messenger to promote cell proliferation and differentiation. By limiting ROS produced via mitochondrial respiration, the quiescent cells not only avoid cellular damage but also maintain the quiescent HSCs population. To support this link between HSCs’ metabolic environment and cell fate, quiescent HSCs have commonly been found in hypoxic environments which prevents oxidative phosphorylation and restricts the cell to relying on anaerobic glycolysis [69,70]. Maintaining quiescent HSCs in hypoxic environments may be a mechanism to separate specific populations of quiescence HSCs from HSCs required for differentiation and/or proliferation.

When HSCs exit quiescence, they must commit to not only proliferate but potentially differentiate into committed progenitor cell lines. Once the cell commits to dividing and/or differentiating the energetic demands of the cell increase forcing the cell to rely more heavily on aerobic metabolism [50]. For instance, when HSCs symmetrically divide to two differentiated cell types, the HSC mother cell was found to rely on fatty acid oxidation (FAO) potentially due to the increased cellular energy demand [71,72]. This is intriguing, since it appears that the metabolic state of HSCs can dictate cell fate. Shifting the metabolic pathway from anaerobic metabolism to aerobic metabolism correlates with increased ROS which propels differentiation into future progenitor cell lines. Once HSCs have committed to differentiation, the path to a mature mast cells becomes one step closer.

### 3.2. Granulocyte/Monocyte Progenitors & Bipotent Basophil/Mast Cell Progenitors

Some HSCs or EMPs will develop into MPPs, then CMPs, then GMPs, and then BMCPs prior to eventually maturing into immature mast cell progenitor cells [60]. Unfortunately, there has been little investigation into the metabolic changes occurring from MPPs to CMPs to GMPs to BMCPs. For some of the unknown mast cell progenitor metabolic environments, other cells with the same cellular signaling occur with more concrete empirical work performed on their metabolic environments. Some of the following work provided here was performed in other cells and is speculative for mast cells. One study suggests that the mitogen-activated protein kinase (MAPK) pathway p38 is required for cell cycle progression and differentiation utilizing glycolysis as the primary metabolic process through these stages of development [73]. Significantly more work needs to be performed on these progenitor cell lines.

The next step in our metabolic journey to mature mast cells is the differentiation step from BMCPs to immature mast cell progenitors. The development to immature mast cell progenitors increases the transcription factor GATA-3 [74]. Studies of GATA-3 in T-cells was found to modulate mitochondrial biogenesis and fitness through transcriptional coactivator peroxisome-proliferator-activated receptor γ coactivator-1α (PGC1α) in response to DNA damage [75]. Activation of PGC1α promotes mitochondrial biogenesis which increases healthy mitochondria and mitochondrial respiration [76]. This may indicate that during differentiation from HSCs to GMPs, there is increased aerobic metabolism leading to DNA and/or cellular damage caused by increased ROS. Upregulation of GATA-3 transcription in the cell may be overcoming the increased damage associated with the increased need for mitochondrial respiration. In addition to increasing the transcription factor GATA-3, the transcription factor CCAAT/enhancer-binding protein alpha (CEBPα) is increased. CEBPα is known to upregulate glucose and lipid metabolism in hepatic and adipose tissue [77]. While studies have not been performed on GMP cells to determine if the same glucose and lipid regulations are taking place, CEBPα is likely contributing to the cellular metabolism at this critical stage. Interestingly, once differentiated into immature mast cell progenitors the CEBPα transcription factor is decreased to further differentiate into mature mast cells. This indicates that different predominant metabolic pathways (aerobic or anaerobic metabolism) are used to drive the cell to further differentiate depending on the progenitor mast cell. This metabolic shift between the two mast cell development stages is a potential area to further explore how these metabolic changes are influencing cell fate decisions. This link between metabolism and cellular fate could be vitally important in offering novel therapeutic targets in diseases such as mastocytosis where there is a massive proliferation of dysfunctional mast cells.

Another transcription factor that is increased to determine cell fate from GMPs to immature mast cell progenitors is HES-1. HES-1 is required for stem cells to differentiate and is positively regulated through increases in ROS indicating potential regulation through cellular metabolism [78]. This implies that aerobic metabolism may be the key metabolic pathway used to differentiate from GMPs to immature mast cell progenitors. The microphthalmia-associated transcription factor (MITF) is increased in immature mast cell progenitor cells and is required for differentiation into fully mature mast cells [58]. MITF has been linked to metabolism by activating transcription of PGC1α that is also activated by the transcription factor GATA-3. This offers two pathways to activate PGC1α when transitioning from GMPs to immature mast cell progenitors.

Currently, there is little known about the cellular metabolism of the different progenitors leading to the mast cell progenitor. The metabolism during these different stages offers an area for future studies to understand the different metabolic changes occurring during each one of these precursors that led to mast cell progenitors. It is likely that cellular metabolism in the mast cell progenitor lines is dynamic and not reliant on one single form of metabolism (i.e., glycolysis, oxidative phosphorylation), similar to what was observed in HSCs. Each one of these cells will undergo specific differentiation, which could be regulated by a distinctive cellular metabolism dictating the ultimate progenitor cell fate as observed in HSCs. Mast cells are unique from many other bone marrow-derived cells because immature mast cells do not fully mature in the bone marrow but rather in the target tissue they localize to following exposure to specific growth factors and cytokines. This requires external stimulus to aid in the differentiation process of mast cells.

### 3.3. Immature Mast Cell

One of the pivotal growth factors responsible for immature mast cell differentiation into mature mast cells is the stem cell factor (SCF), which is also called the steel factor, Kit ligand, and mast cell growth factor [52,53]. SCF is required for mast cell progenitor cells expressing the CD34 antigen and the kit receptor [54]. While other cytokines such as IL-3 are critical for differentiation, SCF is required for human mast cell development. SCF is vital for differentiation of both connective tissue mast cells (CTMCs) and mucosal mast cells (MMCs) which are identifiable by the production of only tryptase or chymase and tryptase together in granules, respectively [79]. Besides SCF playing an essential role in the development and differentiation of mast cells, SCF is required for proliferation, chemotaxis, adhesion, survival, and activation of mast cells [54]. The binding of SCF to the kit receptor activates central intracellular pathways that significantly alter cellular metabolism.

The SCF homodimer binding results in rapid dimerization of the kit receptor and activation of tyrosine kinase causing phosphorylation of the kit receptor catalytic site [80,81]. The phosphorylated sites act as anchor points for adaptor proteins (SH2, Src) recruiting growth factors (Grb2) to the kit receptor [82]. These interactions among others will activate the MAPK signaling pathway in a three-tier phosphorylation cascade. The first tier proteins Ras [82] and Raf1 [82] will become phosphorylated and activate all three families of MAPK; extracellular signal-regulated kinase (ERK1/2 or p42/44), p38, and c-Jun N-terminal kinase (JNK) [54]. The MAP kinase ERK1/2 pathway will activate phosphatidyl inositol-3 kinase (PI3K), which then activates Akt and mTOR driving cell proliferation to progress from the G1 phase to the S phase [83]. Activation of the MAPK signaling pathway leads to the transcription of many genes responsible for cell proliferation, survival, and motility [84]. Mast cell proliferation relies on SCF binding to the kit receptor resulting in activation of PI3K and subsequent activation of Akt and mTOR [85]. SCF has been shown to activate the PI3K/Akt pathway [54,86] likely via activation of the MAPK ERK1/2 pathway. While the downstream effects of activating this pathway are known, the precise metabolic environment has not been fully revealed in mast cells or their progenitors.

The Src family of proteins has been shown to be vital for mast cell proliferation as observed in Lyn deficient mice which display an inability for their mast cells to proliferate [87]. Mast cells require the initial upstream activation of the Src family kinases for mast cell growth [87]. Src activation activates the MAPK JNK pathway propelling mast cell proliferation through activation of mTOR. Activation of mTOR increases glucose metabolism, nucleotide, protein, and lipid synthesis [88] and will upregulate transcription of several glycolytic enzymes shifting the primary metabolism from mitochondrial respiration to glycolysis and PPP [88,89]. Activation of both the MAPK and PI3K/Akt/mTOR signaling pathways force the cellular metabolism to rely on glycolysis and PPP to meet cellular energetic demands and potentially act as directors of cell fate in immature mast cell progenitors.

Studies in other immune cells have indicated that during high proliferation states the cells will utilize glycolysis to meet their high energy demands [90,91]. This mirrors metabolic shifts adopted by cancer cells—termed the Warburg effect or aerobic glycolysis—where the cell favors glycolysis despite having adequate oxygen available for mitochondrial respiration [92]. In other immune cells, activation of both the MAPK signaling pathways ERK and JNK as well as the PI3K/Akt pathway have been shown to increase aerobic glycolysis [93], the pentose phosphate pathway (PPP), and GLUT1 production [91,94]; supporting immature mast cells rely on these metabolic pathways to mature into a mature mast cell. In addition to the MAPK and PI3K/Akt/mTOR pathways, SCF activates phospholipase Cγ (PLCγ) [95] and protein kinase Cα (PKCα) [96]. PLCγ has been shown to activate mTOR independent of the conventional PI3K/Akt pathway using the DAG/PKC signaling in B cells [97]. PKCα has been correlated with cell cycle commitment in T cells [98]. This method to facilitate cell proliferation may apply to mast cells too. No research has been performed thus far on the metabolic functions taking place due to increased PLCγ in mast cells. Further research is required to elucidate the precise metabolic pathways used during differentiation from a mast cell progenitor to a mature mast cell. The majority of our current knowledge on the metabolic effects these signaling pathways induce was not performed using mast cells which may not be representative of mast cell biology. Further work needs to be performed directly on mast cell progenitors and mature mast cells to understand the role of metabolic environment on mast cell development.

### 3.4. Summary of Metabolic Demands during Mast Cell Development

There is significant room for more research regarding the metabolism occurring in developing mast cells. The current research only scratches the surface revealing changes to mast cell progenitor metabolism. Even when analyzing the cellular metabolism of HSCs, where the most research into the metabolic activity has been performed, there are still many questions left unanswered. Many of the intermediate progenitor cell lines from HSCs to mature mast cells have no research on their metabolism. The metabolic functions occurring in these progenitor cell lines are vitally important for proper cellular differentiation and proliferation. Understanding the metabolic environments present that influence proliferation and/or differentiation could offer novel drug targets for mast cell diseases. Mast cells are at the center of many diseases classified into primary (e.g., mastocytosis), secondary or reactive (e.g., autoimmune disorders, allergies, inflammatory diseases, MCAS), and idiopathic disorders (i.e., unknown cause) [99]. These wide-ranging disorders affect a large population of people with many having little to no treatment options. Many of these disorders can potentially be influenced through research on the impact of metabolism on mast cell development. One example is during mastocytosis, where there is a massive proliferation of dysfunctional mast cells leading to increases in activation, causing symptoms that range from discomfort to life threatening anaphylaxis [100]. Currently there are minimal treatment options for this disease. Developing novel therapeutic targets for mast cell differentiation via metabolism could be a mechanism to mitigate both the inappropriate proliferation of mast cells and the negative health effects caused by mastocytosis. This area of research would also likely offer a wealth of information on the idiopathic disorders, where currently there is no known cause for the mast cell related disorder. Some of these disorders could be a result of aberrant metabolic environments present during mast cell development ultimately leading to the disease state. By better understanding the interplay between mast cell metabolism and proliferation/differentiation, novel therapeutic drug targets and better diagnosis techniques could be identified to mitigate and/or eliminate the adverse effects of many mast cell disorders.

In addition to understanding mast cell progenitor metabolism, work needs to be performed on the metabolic environments between the two main phenotypes of mast cells, MMCs and CTMCs. To date, there has been no work to show if any metabolic differences exist between the two types of mast cells or their routes for development. With the clear potential ROS has on driving cellular proliferation and/or differentiation, more work needs to be performed on ROS and their link to mast cell development. Overall, there is a wealth of information waiting to be discovered for metabolism during mast cell development.

## 4. Concluding Remarks

Cellular energy metabolism is an important element of mast cell’s ability to appropriately develop into mature resident tissue cells and properly function once localized in the target tissue. Research shows that different sources of activation result in distinct shifts of metabolism with the role of oxidative phosphorylation and glycolysis explored in IgE mediated activation and glycolysis explored in non-IgE activation. A central role of ATP production and utilization was also apparent; however, there were some confounding results as ATP is also shown to activate mast cells independently through purinergic receptors. Blocking metabolic pathways and observing a decrease in several mechanisms of activation shows that these energy metabolic changes are necessary for function and precede any exocytosis pathway. Further, these changes in activation occurred without changes in intracellular ATP, indicating that metabolic intermediates may be involved in processes not solely related to producing ATP. This provides the potential to target these pathways in order to develop early diagnosis techniques and novel therapeutics for mast cell dysfunction related symptoms in sensitive populations such as those suffering from mast cell activation syndrome (MCAS).

Energy metabolism was also shown to be modulated in progenitor cell populations during the development of mast cells. Overall, significantly less research has been performed on this topic but it is clear that different stages of maturation involve emphasis on either glycolysis, oxidative phosphorylation, or even fatty acid oxidation. This indicates varying energy requirements are present depending on the level of development and perhaps these energy requirements or ROS concentrations are what dictate development and cell fate. If metabolism can be elucidated during these steps, it could further provide therapeutic opportunities for diseases occurring from inappropriate mast cell proliferation and development such as mastocytosis. Overall, cellular energy metabolism is a basic but essential area of research that must be explored further not only to fully understand mast cells but also to deal with numerous pathologies that occur from mast cells not functioning properly.

## Figures and Tables

**Table 1:** Summary of studies exploring energy metabolism changes in IgE mediated mast cell activation.

Mast Cell Source/Cell Line	Activation Phase/Endpoint	Metabolic Pathway Explored	Metabolic Endpoint	Year/Ref
Peritoneal	Early phase (histamine)	Glycolysis	Increased lactate export	1974 [23]
Peritoneal	Early phase (histamine)	Oxidative phosphorylation	Increased oxygen consumption	1968 [25]
Peritoneal	Early phase (histamine)	ATP	Increased ATP production	1975/1979 [26,27]
BMMC	Early phase (β-hexosaminidase)	Glycolysis	Increased D-glucose=increased activation	2010 [28]
BMMC	Late phase (LTC4 and PGD2)	Glycolysis	Increased D-glucose=increased activation	2010 [28]
RBL	Early phase (β-hexosaminidase)	Glycolysis/ Oxidative phosphorylation	Decreased M2-type pyruvate kinase	2008 [29]
HMC/LAD2	Early phase (β-hexosaminidase)	Glycolysis	Inhibit Aldolase A=increased lactate export	2010 [30]
LAD2/RBL	Early phase (β-hexosaminidase)	Oxidative phosphorylation	Mitochondria translocated to exocytosis site	2011 [31]
LAD2/RBL	Late phase (TNFα)	Oxidative phosphorylation	Mitochondria translocated to exocytosis site	2011 [31]
BMMC/RBL/LAD2	Early phase (β-hexosaminidase)	Oxidative phosphorylation	Increased STAT3-dependent oxygen consumption	2014 [32]
BMMC/RBL	Early phase (β-hexosaminidase)	Oxidative phosphorylation	Inhibit pyruvate dehydrogenase=decrease respiration/activation	2017 [33]
BMMC/RBL	Late phase (TNFα and IL-6)	Oxidative phosphorylation	Inhibit pyruvate dehydrogenase=decrease respiration/activation	2017 [33]

**Table 2:** Summary of studies exploring energy metabolism changes in non-IgE mediated mast cell activation.

Mast Cell Source/Cell Line	Agonist	Activation Phase/Endpoint	Metabolic Pathway Explored	Metabolic Endpoint	Year/Ref
Peritoneal	Compound 48/80	Early phase (histamine)	Glycolysis	Increased lactate export	1974 [23]
Peritoneal	Compound 48/80	Early phase (histamine)	Pentose Phosphate Pathway	Increased conversion of 14C1-/14C6 to 14CO_2_	1985 [37]
Peritoneal	Compound 48/80	Early phase (histamine)	ATP	Increased ATP	1975 [26]
Peritoneal	Compound 48/80	Early phase (histamine)	Glycolysis	Increased glycolysis=increased ATP	1991 [38]
BMMC	IL-33	Late phase (TNFα, IL-6, CCL2)	Glycolysis/Oxidative phosphorylation	Increased extracellular acidification rate (ECAR) and oxygen consumption rate (OCR)	2018 [39]
BMMC	IL-33	Late phase (TNFα, IL-6, CCL2)	Glycolysis	Inhibit glucose uptake (2-DG)=decrease cytokines	2018 [39]
In vivo, *C57Bl/6*	IL-33	Neutrophil recruitment	Glycolysis	Inhibit glucose uptake (2-DG)=decrease cell recruiting	2018 [39]
BMMC/Peritoneal	LPS	Late phase (IL-6 and CCL2)	Glycolysis	Decrease lactate export=decrease cytokines	2019 [40]

## Data Availability

Not applicable.
